# A monopole antenna with cotton fabric material for wearable applications

**DOI:** 10.1038/s41598-023-34394-3

**Published:** 2023-05-05

**Authors:** Ayman Ayd R. Saad, Walaa M. Hassan, Ahmed A. Ibrahim

**Affiliations:** 1 Kosseir Radio, Telecom Egypt, Kosseir, 84712 Egypt; 2 Electronics Research Institute, El-Nozha El-Gadida, Cairo, 11843 Egypt; 3 Electronics and Communications Engineering Department, Minia University, Minia, 61111 Egypt

**Keywords:** Electrical and electronic engineering, Biomedical engineering

## Abstract

A monopole antenna operated at 2.45 GHz and embedded with artificial magnetic conductor (AMC) for wearable communication systems is investigated in this article. The proposed antenna is composed of a metalized loop radiator with a coplanar waveguide microstrip feedline which is affixed on a cotton fabric material substrate. As well, a cotton-based AMC surface is utilized to eliminate the body’s absorbed radiation and enhance the gain of the antenna. It is composed of 5 × 5 array unit cells etched with I-shaped slots. Using this configuration, simulations show that the specific absorption rate (SAR) level was significantly reduced. Considering flat and rounded body parts, it was found that the SAR values averaged over 10 g at a distance of 1 mm away from the tissues model were only 0.18 W/kg and 0.371 W/kg, respectively. Additionally, the antenna gain was improved up to 7.2 dBi with an average radiation efficiency of 72%. Detailed analysis with experimental measurements of the cotton-based antenna for different operation scenarios is introduced. The measured data show a good correlation with the electromagnetic simulation results.

## Introduction

Nowadays, WBANs are applied in healthcare and medical applications^1–3^. In WBANs systems, wearable antennas are vital components used for communication near the human body^4–6^. This challenging role is reflected in the considerations that take when designing such types of antennas. One of these considerations is the influence on the antenna resonance behavior due to the loading effect of the high permittivity body tissue^7,8^. On the other hand, in antenna design choosing flexible materials have to be considered to be utilized close to the rounded parts of the human body. Several kinds of wearable antennas based on flexible materials are studied and investigated by researchers such as textile^9^, flexible substrate^10^, dielectric resonators^11^, polyimide^12^, polydimethylsiloxane^13^, paper^14^, and Kapton^15^. Among these materials, textiles are preferred due to their lightweight and high flexibility in integration with clothing^16^. However, the implementation process of wearable antennas using textile fabrics as substrates is more difficult compared to the use of conventional substrates^17^.

As wearable antennas operate near the human body, their radiation can cause damage to body tissues. This effect is examined by evaluating the SAR level by considering a specific part of the human body. To reduce the health risks introduced by the wearable antennas, the SAR values should be below the regulated level^18,19^. In the literature, several techniques have been reported to reduce the body's absorbed radiation and, consequently, minimize the SAR level^20–36^. One of the common techniques is using a reflector below the antenna. Different structures have been utilized as reflectors such as high impedance surfaces (HISs)^20^, electromagnetic bandgap (EBG) structures^21–26^, and artificial magnetic conductor (AMC) surfaces^27–36^. These structures can increase the antenna gain and help significantly reduce its overall profile compared to the use of a traditional perfect electric conductor (PEC) structure.

Among the reported reflector structures, AMC surfaces have been widely used for backing wearable antennas^27–36^. In^27^, a flexible reconfigurable antenna backed with an AMC surface that worked at 2.4/3.3 GHz was introduced. Considering a human leg model, the evaluated SAR values do not exceed 0.29 W/kg for both operating bands with increasing in the antenna gain by 3.6 and 2.4 dB, respectively. In^28^, a Yagi–Uda antenna built on a latex substrate and combined with an AMC surface was presented to operate at 2.4 GHz. Single- and double-layered AMC surfaces were used to minimize the peak SAR level to 0.714 W/kg and increase the peak gain up to 1.8 dBi. In^30^, the performance of a wearable antenna over an AMC surface based on using a stretch conductive fabric was investigated. The design enabled the antenna to cover both WiFi and the 4G long-term evolution (LTE) frequency bands.

A textile antenna with AMC surface for WLAN/WBAN applications was reported in^32^. The integrated geometrical configuration reduced the SAR value and improved the gain to 0.0721 W/kg and 2.42 dBi, respectively. In^33^, a flexible AMC surface was used as a reflector. It provides stable performance and reduction in SAR level. In the reported study, the effect of crumpling of the integrated antenna was analyzed. In^34^, a flexible antenna backed with an AMC ground plan and operates at 2.4 for telemedicine applications is reported. Utilizing the AMC plane provides a 3.7 dB increase in gain, in addition to a 64% reduction in SAR value. A design of compact wearable antennas resonated around 2.65 GHz is reported in^35^. The backward radiation was reduced using a metasurface recognized as an AMC plane and modeled with a CRLH transmission line operated at negative modes. The peak SAR value of the proposed antenna is 1.25 W/kg for a 5 mm gap from the human body with a real gain of 0.82 dBi. A dual-band 1.57/2.45 GHz wearable antenna with AMC structures is discussed in^36^. The antenna has a SAR level lower than 0.12 W/kg and a gain value of about 1.9 dBi at the two bands.

In this work, a design of a cotton-based wearable antenna over an AMC surface is proposed for 2.45 GHz applications. The integrated antenna adopts cotton fabric as a substrate to ease integration into clothes. In the CST Microwave Studio, the performance and radiation results demonstrated that the antenna provides excellent on-body performance and achieved SAR values below the regulated limit. Detailed discussions on antenna designs with comparative analysis with recent relevant work were presented. Based on the numerical model, the proposed antenna and the AMC surface were fabricated, integrated, and tested. Good agreements between simulated results and measured data were observed. In the end, we can conclude the contributions of the work as,

- The proposed antenna is fabricated on textile material to achieve lightweight and high flexibility when integrated with clothing.
- The deformation of the integrated antenna was analyzed in free space as well as when it was placed in the vicinity of the human body, indicating its good suitability for operation when bent at both the *x*-axis and the *y*-axis.
- The integrated antenna has a realized gain of 7.2 dBi with average radiation and total efficiency of 72% and 60%, respectively.
- The integrated antenna has a low SAR level averaged over 10 g at a distance of 1 mm away from the tissues model where only 0.18 W/kg and 0.371 W/kg, respectively.

## Antenna and AMC surface

### Single-band wearable antenna

**Figure 1:**
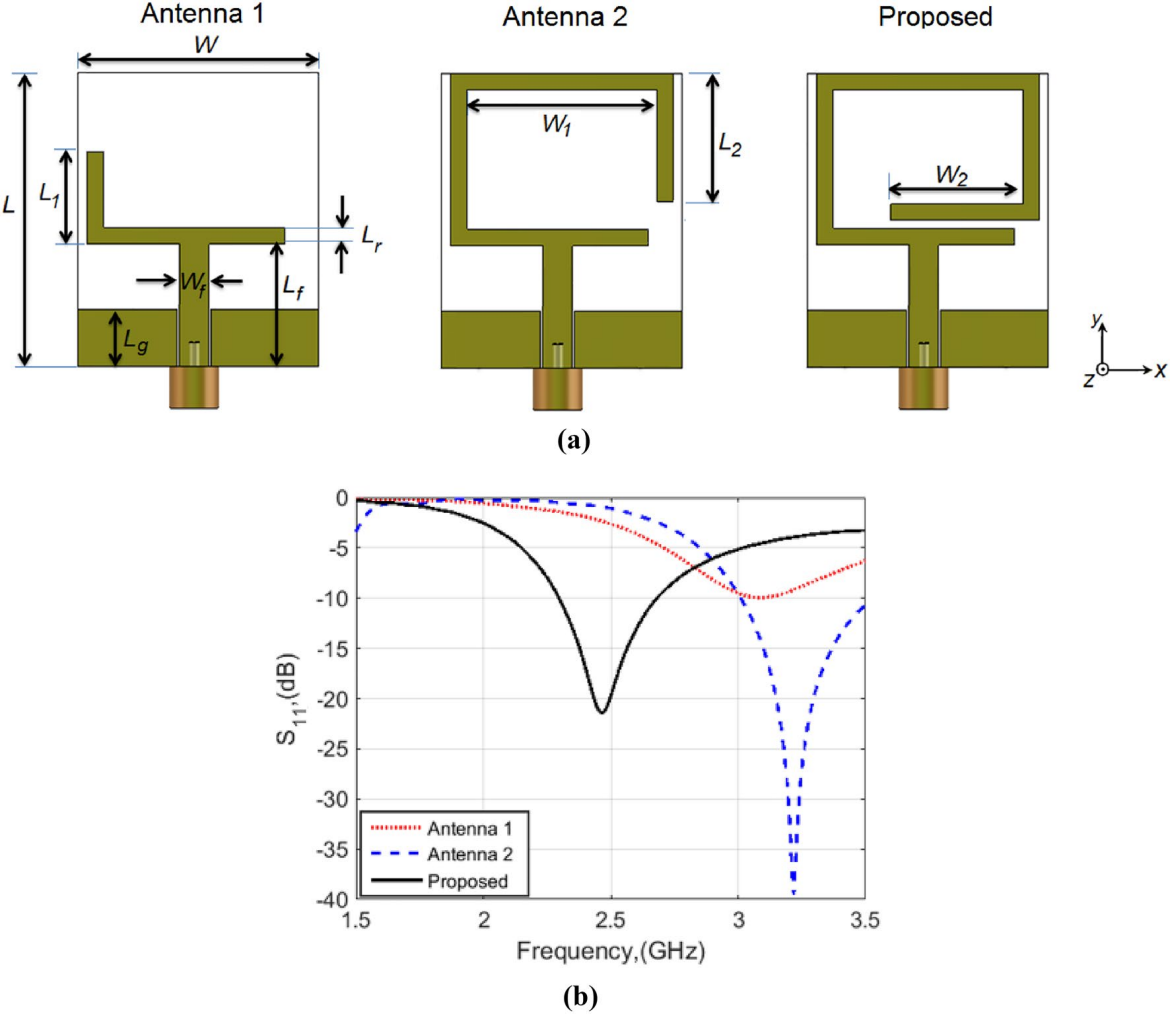
(**a**) The design steps of the proposed wearable antenna (*L* = 36 mm, *W* = 30 mm, *L*_1_ = 10 mm, *L*_2_ = 16 mm, *W*_1_ = 23 mm, *W*_2_ = 16 mm, *L*_*r*_ = 2 mm, *L*_*f*_ = 15 mm, *W*_*f*_ = 3.5 mm, *L*_*g*_ = 7 mm). (**b**) Simulated |S_11_| responses versus frequency.

The design steps of the proposed wearable monopole antenna are depicted in Fig. 1a. The antenna is built on a 0.9 mm single-layer cotton fabric with a relative permittivity of *ε*_*r*_ = 1.7. A metallic layer is manually attached to the fabric-base substrate to form the antenna radiator and ground plane. As illustrated in the figure, Antenna 1 consists of an L-shaped radiator fed by a 50Ω co-planar waveguide (CPW) line as the first step of the design. With this configuration, a weak resonance around 3.1 GHz is achieved, as shown in Fig. 1b. To enhance the antenna performance, Antenna 2 is designed, where the radiator is extended to a C-shaped. Figure 1b shows that Antenna 2 can operate around 3.3 GHz with good matching performance. Finally, to tune the antenna resonance to the desired frequency band around 2.45 GHz, an antenna with a loop radiator is designed. The optimized design parameters are listed in the caption of Fig. 1, which shows that the proposed antenna can resonate around 2.45 GHz with a bandwidth (BW) extended from 2.29 to 2.69 GHz (10 dB return loss).

### Single-band AMC surface design

**Figure 2:**
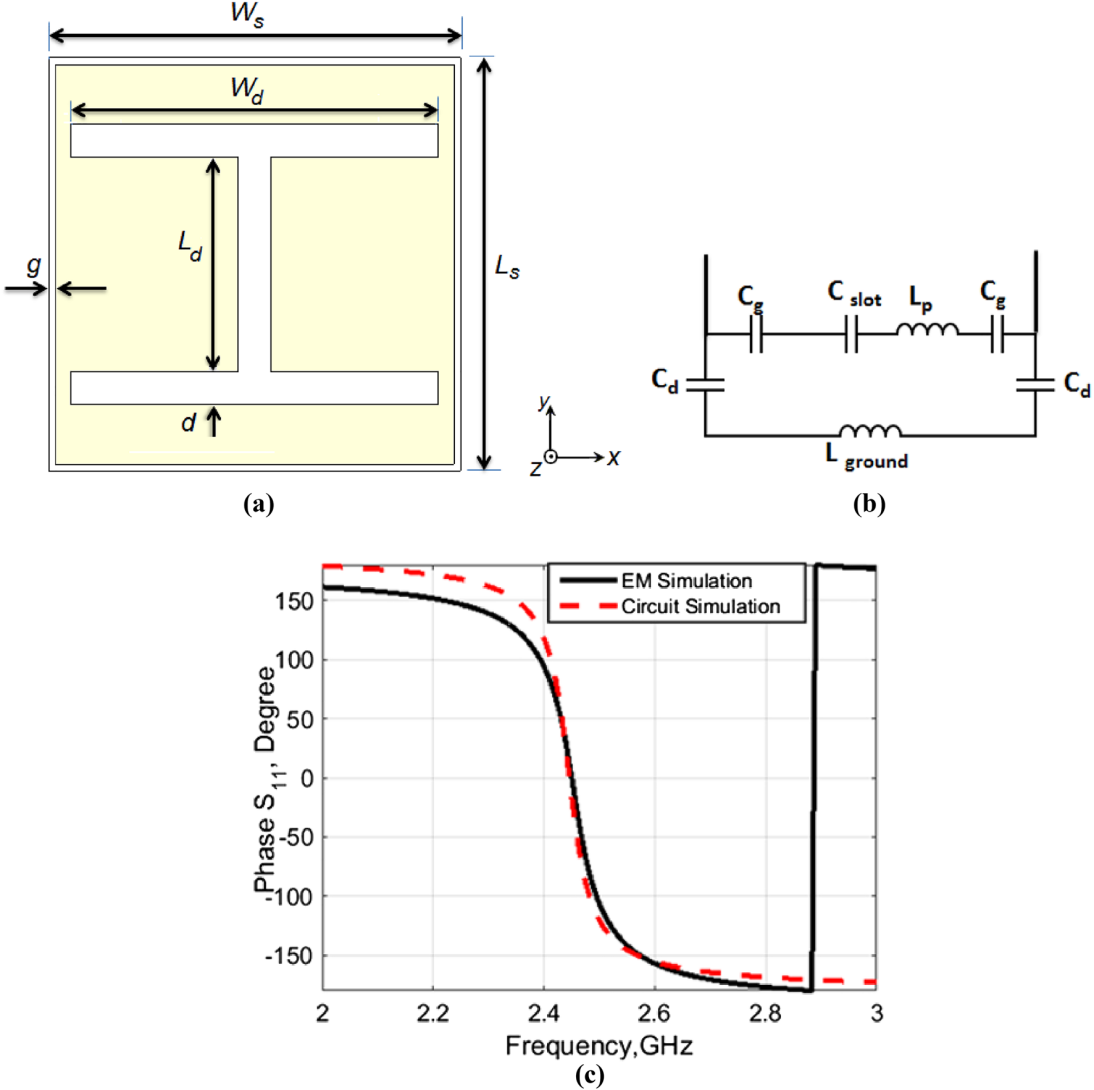
(**a**) Proposed square patch unit cell of the AMC surface. (**a**) Geometrical configuration (L_s_ = W_s_ = 24.5 mm, W_d_ = 22.3 mm, L_d_ = 13 mm, d = 2 mm, g = 0.25 mm). (**b**) Unit cell equivalent circuit model (C_g_ = 2.942 pF, C_d_ = 2.452 pF, C_slot_ = 4.85 pF, L_p_ = 5.842 nH, L_ground_ = 1.42 nH). (**c**) Simulated reflection phase responses versus frequency.

**Figure 3:**
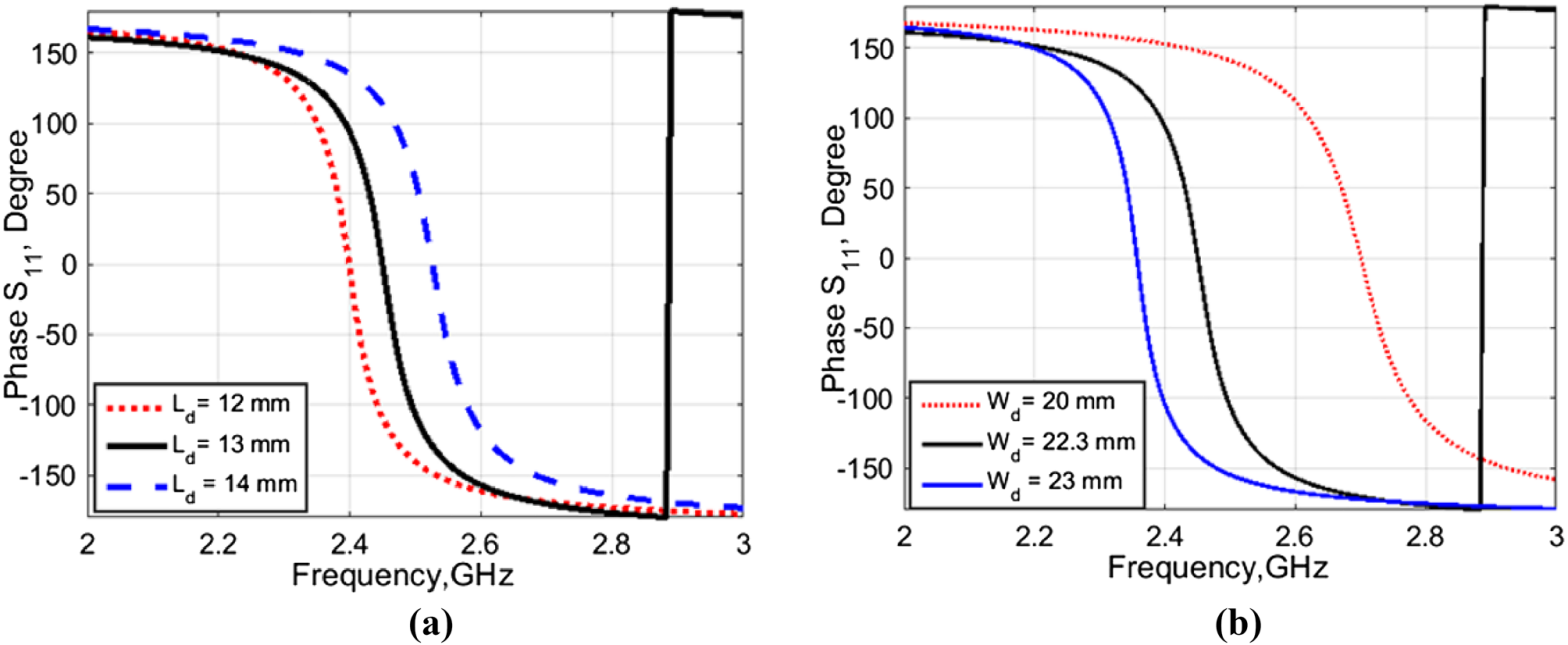
Effect of the length, L_d_ (**a**) and width, W_d_ (**b**) of the I-shaped slot on the phase response.

**Figure 4:**
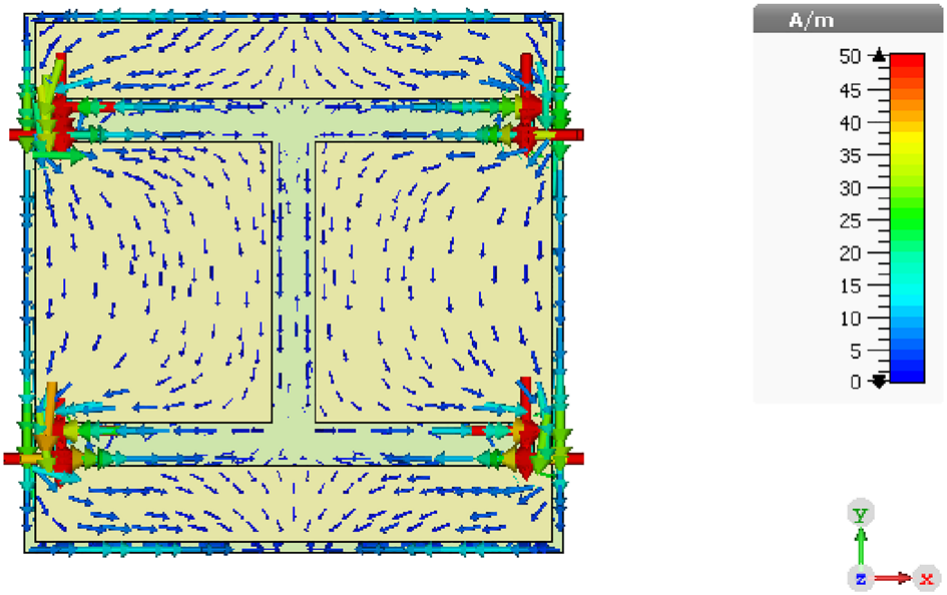
Illustration of surface current distribution on the proposed square patch unit cell of the AMC surface at 2.45 GHz.

To eliminate the body's absorbed radiation and enhance the antenna gain, the proposed antenna is placed on an AMC surface to reduce the overall profile of the entire structure compared to the use of the PEC surface. Such surfaces operate as inductor–capacitor (L–C) tank circuit at the resonance frequency and achieve HIS. The proposed AMC surface is designed to achieve in-phase reflection at the antenna’s resonance frequency of 2.45 GHz. It was built on a double-compacted layer of cotton fabric with a thickness of 1.8 mm. It consists of 25 square patch unit cells (5 × 5 array) with I-shaped slots, which occupy a whole area of 122.5 × 122.5 mm^2^. The geometrical configuration of the proposed unit cell and its equivalent circuit are depicted in Fig. 2a,b, respectively. The ADS software is used to model the equivalent circuit, where the ground plane, the substrate, the I-shaped slot, the rectangular patch, and the gap, *g* is modeled as *L*_*ground*_, *C*_*d*_, *C*_*slot*_, *L*_*P*_, and *C*_*g*_, respectively^37,38^. The optimized lumped element values are displayed in the caption of Fig. 2. The outcome of the circuit simulation is compared with that of the EM simulation and shown in Fig. 2c. A good tendency between the two outcomes is observed. The in-phase (− 90° to + 90°) frequency range is 2.4–2.5 GHz with 0° phase at 2.45 GHz. The effect of the length, *L*_*d*_ and width, *W*_*d*_ of the I-shaped slot on the phase response is illustrated in Fig. 3a,b, respectively. It can see that, the 0° phase is shifted up with the increase of the *L*_*d*_, while it shifted down with the increase of the *W*_*d*_. The optimized values of the *L*_*d*_ and *W*_*d*_ are 13 and 22.3 mm, respectively. The surface current density distribution at 2.45 GHz is examined in Fig. 4. As can be seen, the currents are distributed around the edges of the slot.

## AMC-backed antenna

**Figure 5:**
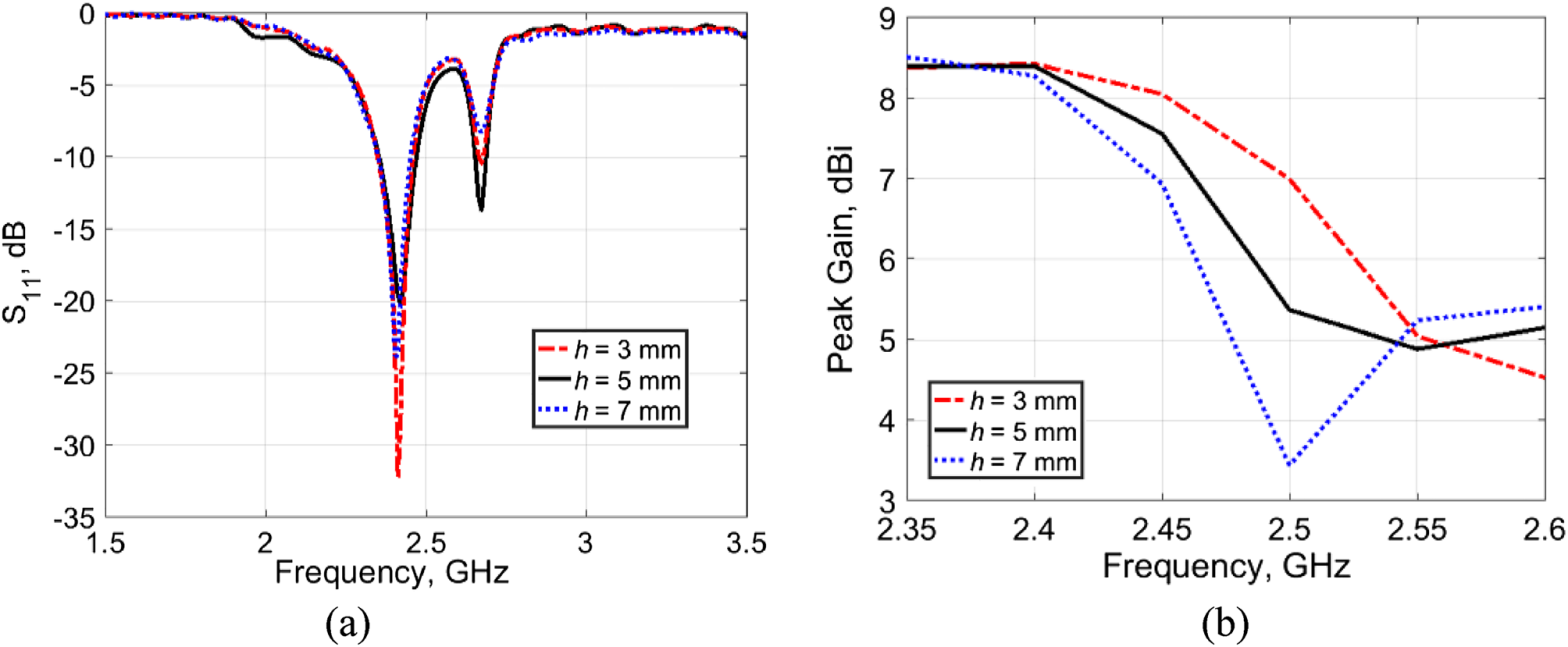
Effects of the spacing of the AMC surface on antenna performance with 5 × 5 array size. (**a**) |S_11_| response. (**b**) Peak gain.

**Figure 6:**
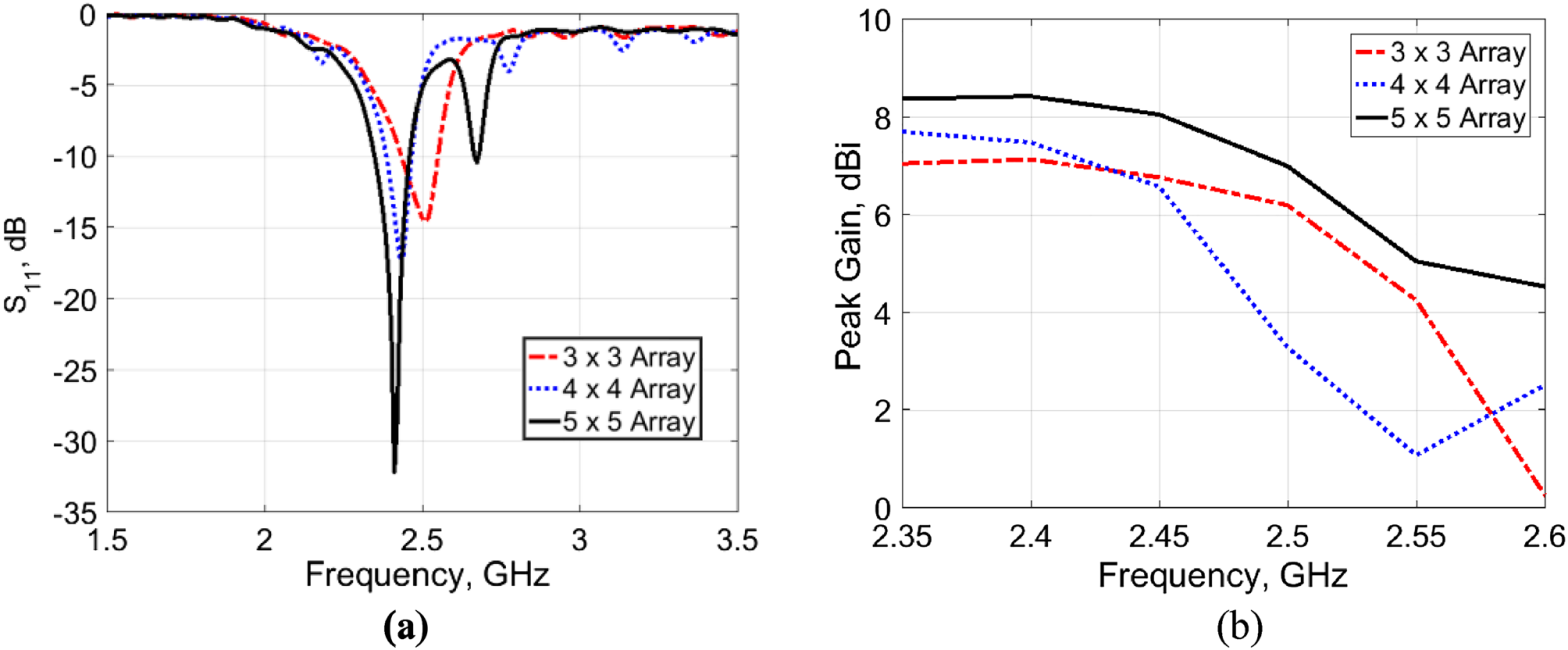
Effects of the size of the AMC surface on antenna performance with 3 mm spacing. (**a**) |S_11_| response. (**b**) Peak gain.

In this section, the performance of the single-band wearable antenna over the designed AMC surface is investigated. Two significant parameters that affect the antenna’s performance were subjected to study, the spacing of the AMC surface below the antenna and its array size. Parametric studies were carried out to adopt these parameters. Figure 5a,b show the influence of three separation distances, namely *h* = 3, 5, and 7 mm on the antenna performance in terms of |S_11_| response and peak gain, respectively. The corresponding effects of varying the AMC array size are shown in Fig. 6a,b, respectively. From the figures, it can be observed that with a spacing of 3 mm between the antenna and a 5 × 5 AMC array, strong resonance occurs at 2.45 GHz with a peak gain of 8 dBi. Also, it can be observed that the AMC surface caused a weak resonance at 2.7 GHz. This resonance can be reduced by increasing the space between the antenna and the AMC layer, but in return increasing the overall antenna size.

**Figure 7:**
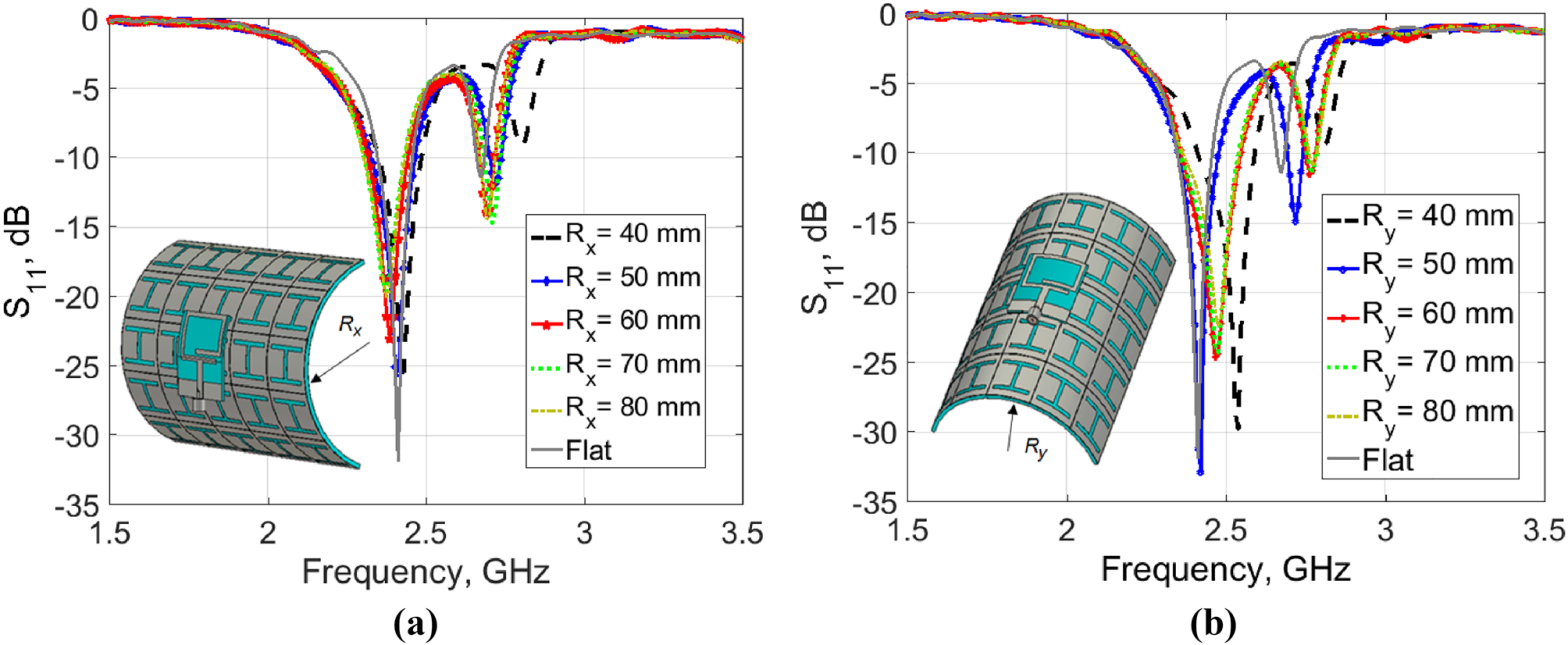
The |S_11_| response with different curvature radii. (**a**) Along x-axis. (**b**) Along y-axis.

In practical applications, the wearable antenna is expected to be bent during the operation. To ensure the reliability of the designed antenna for such a scenario, the integrated antenna was subjected to structural bend along both the *x*-axis (in the *L* direction) and the *y*-axis (in the *W* direction). Five different curvature radii along each of the *x*-axis (*R*_*x*_) and *y*-axis (*R*_*y*_), namely 40, 50, 60, 70, and 80 mm were separately studied. These values are reasonable representations of curvature radii of various rounded positions of the adult human body. The simulated |S_11_| responses versus frequency for both bending scenarios are displayed in Fig. 7a,b. In each scenario, the bending radius along one axis was changed and kept zero for the other (i.e. flat). As can be seen, the impedance BW of the bent AMC-backed antenna isn’t changing which indicates that the antenna has good suitability in such scenarios for operation. However, a slight upper-frequency shift appears for the bending scenario at the y-axis, especially with a curvature radius of 40 mm.

## Performance of the AMC-backed antenna on the human body

In this section, the performance of the proposed wearable antenna backed by the designed AMC surface is investigated when considered for operation in the vicinity of human tissues at a distance of 1 mm. To simulate the antenna performance, the Hugo voxel-based body model presented in CST Microwave Suite was used. The Hugo model is an inhomogeneous human model built from 32 tissues. Each tissue has material properties that reflect the anatomical human tissue properties. In this study, the Hugo model allows the determination of the loading effect of the human body on antenna performance and a detailed analysis of the SAR distributions.

### Antenna characteristics

**Figure 8:**
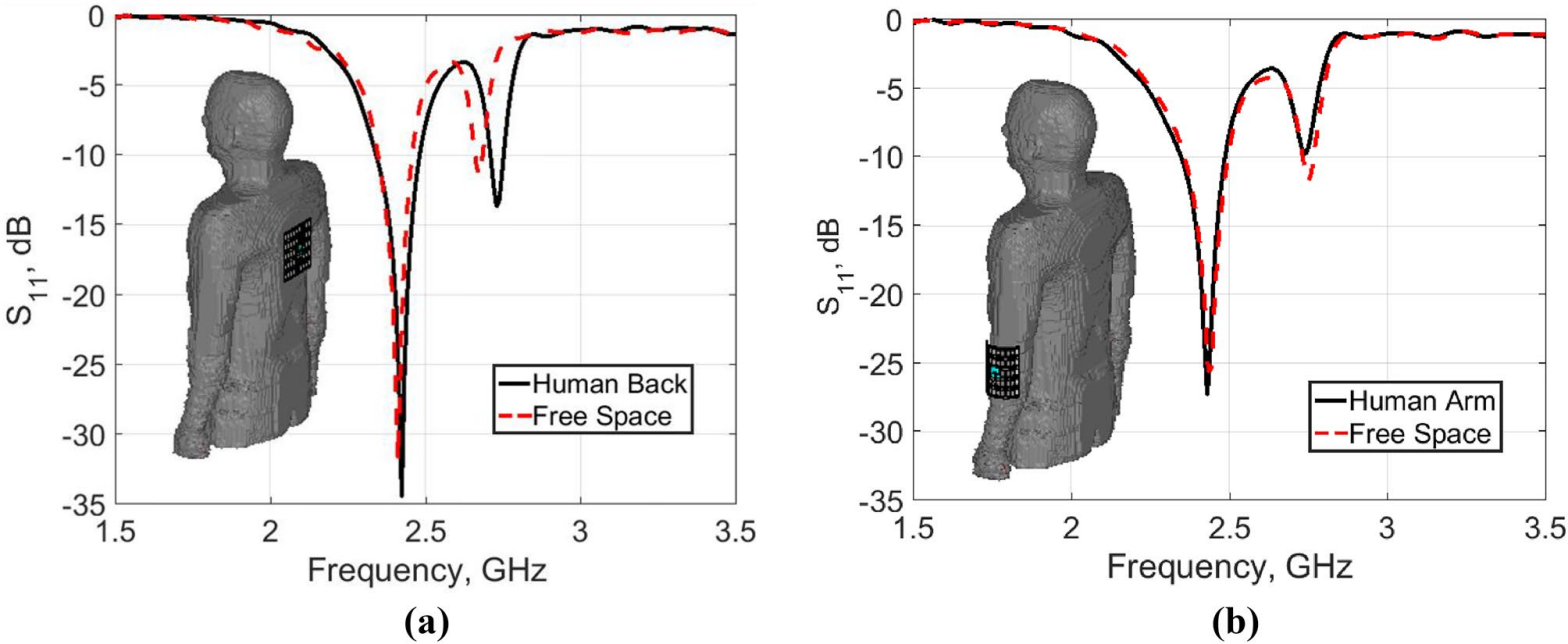
The |S_11_| response of the antenna evaluated in free space in comparison to that of body loading for (**a**) Flatback. (**b**) Arm of radius 50 mm.

**Figure 9:**
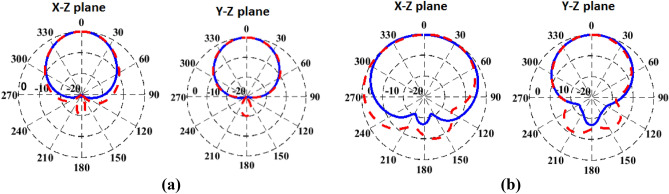
The radiation patterns characteristics of the AMC-backed antenna at 2.45 GHz evaluated in free space (dashed) in comparison to that of body loading (solid) for (**a**) flatback. (**b**) Arm of radius 50 mm.

The antenna characteristics for flat and rounded body loading were studied. The evaluation was performed in terms of |S_11_| response and radiation characteristics. Figure 8a,b show with a good agreement the |S_11_| responses evaluated in free space in comparison to that of body loading for a flat back and rounded arm of radius 50 mm, respectively. The corresponding far-field radiation patterns are illustrated in Fig. 9. Slightly effect on antenna performance is observed when loaded onto the human body.

**Figure 10:**
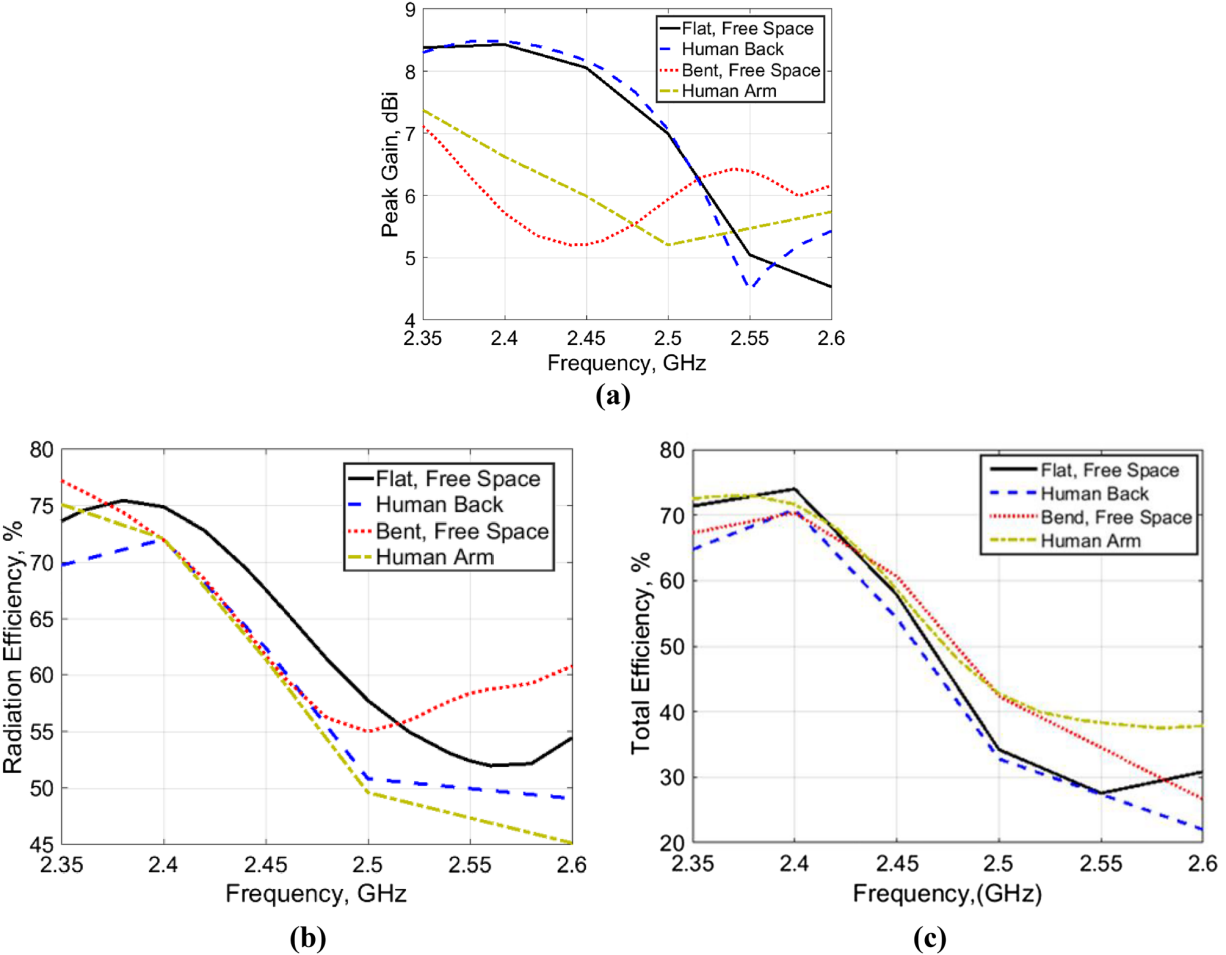
Simulated radiation characteristics of the antenna in free space and when placed close to the human body model. (**a**) Peak gain, (**b**) radiation efficiency. (**c**) Total efficiency.

Figure 10 shows the radiation characteristics of the AMC-backed antenna in free space in comparison to that of body loading. The results show that the peak gain of the flat AMC-backed antenna in free space and when attached to the human back model is almost not affected, while a slight effect of 0.85 dB occurs in the peak gain of the bent antenna (*R*_*y*_ = 50 mm) when attached to the human arm. The radiation efficiency is almost stable and varies from 70 to 72% for all cases. As well the total efficiency is almost stable and varies around 60% for all cases.

### SAR evaluation

The SAR level is used to evaluate the amount of RF (radio frequency) energy absorbed by the human body. The Council of the European Union recommended a SAR value of 2 W/kg averaged over 10 g of tissues^19^. The SAR level is expressed as^26^:

1 $${\text{SAR}} = \sigma /\rho |E|^{2}$$

where σ is the conductivity of the tissue in S/m, *ρ* is the mass density of the tissue in kg/m^3^, and *E* is the total RMS electric field strength in V/m.

**Figure 11:**
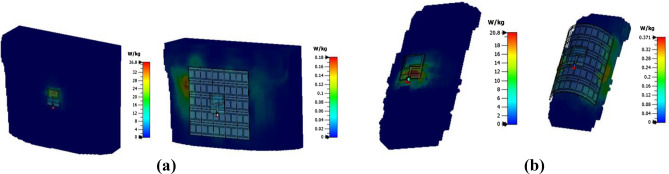
SAR values evaluated over 10 g of tissues of the proposed antenna alone (left) and over the AMC surface (right) at 2.45 GHz on (**a**) flatback. (**b**) Arm of radius 50 mm.

The SAR distribution of the proposed wearable antenna system is evaluated, considering flat (human back) and rounded body (a human arm of radius 50 mm) models. Figure 11a shows the calculated 10 g averaged SAR for the flat antenna at 2.45 GHz. It can be seen that the calculated SAR value for the AMC-loaded antenna is 0.18 W/kg compared to 36.8 W/kg for the flat standalone antenna. The same scenario is shown in Fig. 11b for SAR distribution along the human arm. It can be seen that the calculated SAR value for the AMC-loaded antenna is 0.371 W/kg compared to 20.8 W/kg for the bent standalone antenna. From this evaluation, it can be seen that the SAR level is significantly reduced when the AMC reflector surface is used.

## Design implementation and results

**Figure 12:**
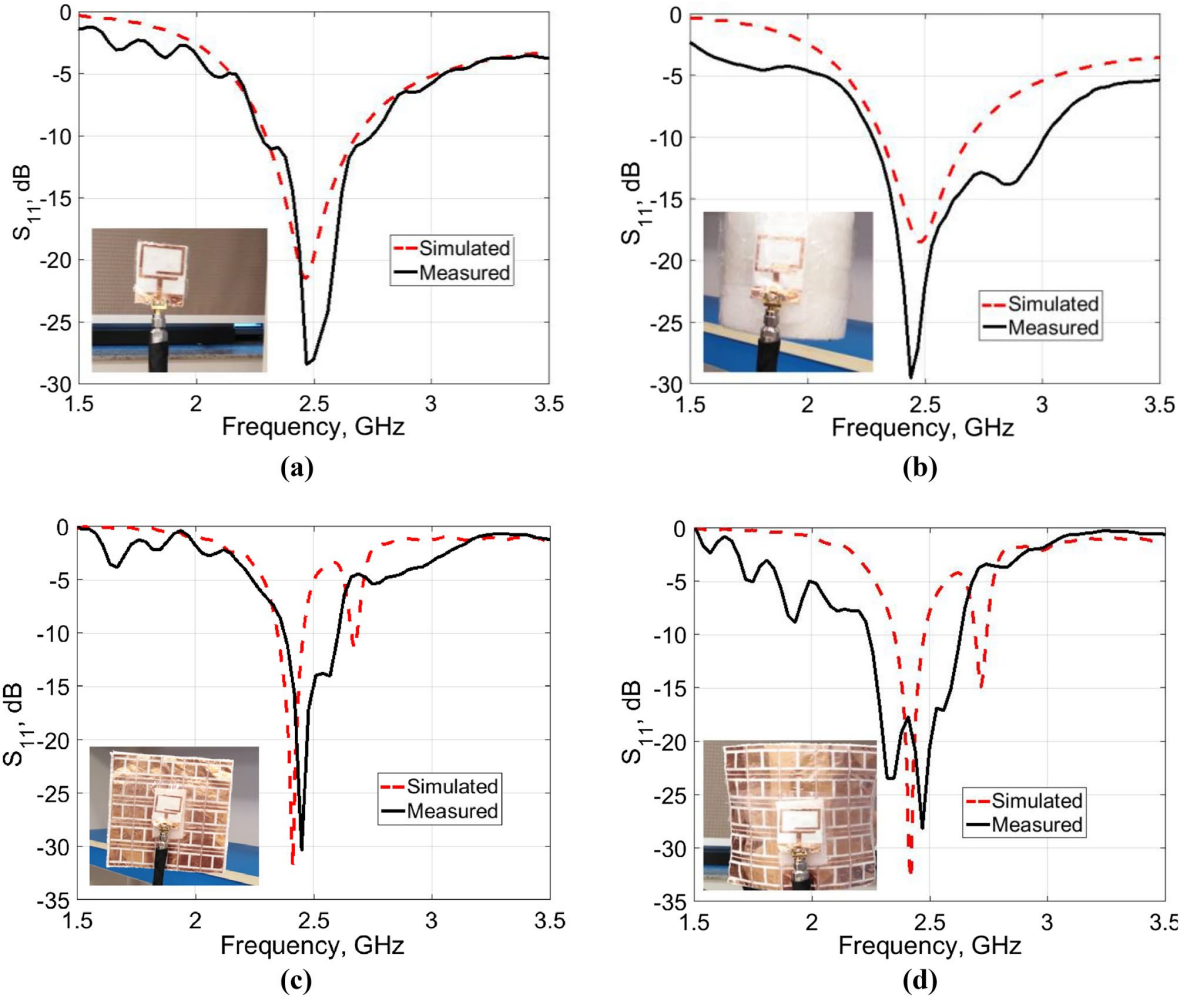
Simulated and measured |S_11_| responses of the proposed wearable antenna for different design cases. (**a**) Flat antenna. (**b**) Bent antenna (*R*_*y*_ = 50 mm). (**c**) Flat AMC-backed antenna. (**d**) Bent AMC-backed antenna (*R*_*y*_ = 50 mm).

In order to examine the practical performance of the proposed wearable antenna system, prototypes were fabricated and subjected to measurement. The proposed antenna and the AMC surface were etched on a conductive layer of 0.035 mm thickness glued to a single layer and a double-compacted layer of cotton fabrics, respectively. The prototype performances were measured through an Agilent N9918A vector network analyzer, where a 50-Ω SMA (SubMiniature A) connector was used to feed the antenna. A comparison of simulated and measured |S_11_| responses of the proposed antenna is given in Fig. 12 for different design cases, the antenna alone in flat and bent states and over the AMC surface for both states. For bending analysis, the antenna was wrapped around a foam cylinder of radius *R*_*y*_ = 50 mm, corresponding to the approximate size of an adult human arm. It can be seen from Fig. 12 that the proposed antenna system can resonate around 2.45 GHz in all cases, with a good agreement between the simulated and measured frequency responses. The measured impedance BW equals 510 and 700 MHz for the antenna alone in flat and bent states, respectively, whereas equals 230 and 370 MHz for the AMC-backed antenna in flat and bent states, respectively, which is suitable for medical applications allocated at this band.

**Figure 13:**
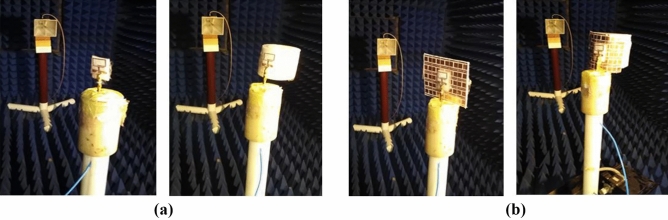
Antenna prototypes under test in an anechoic chamber. (**a**) Excluding AMC. (**b**) Including AMC.

**Figure 14:**
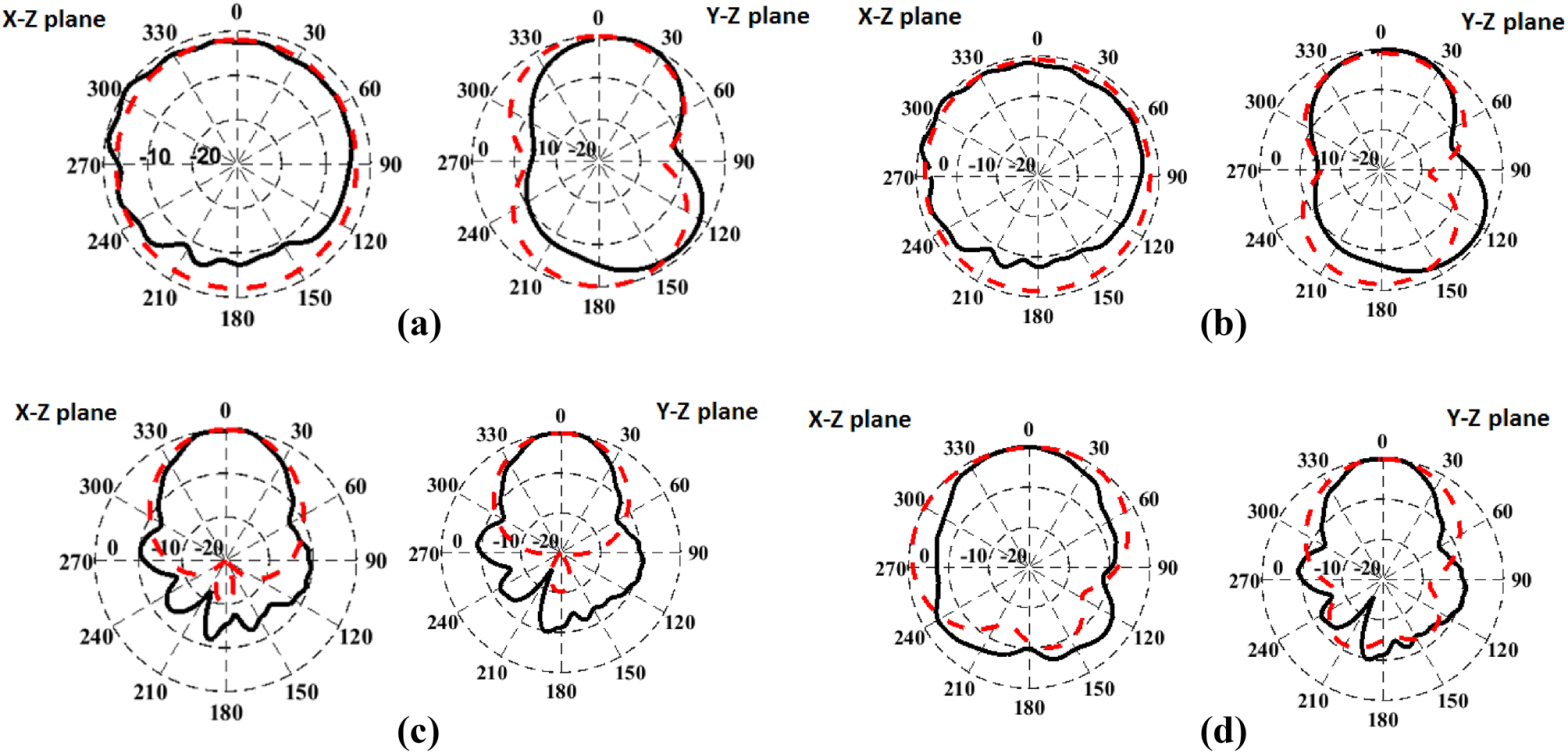
Simulated (dashed line) and measured (solid line) radiation patterns of the proposed antenna system in the *x–z* plane and *y–z* plane at 2.45 GHz. (**a**) Flat antenna. (**b**) Bent antenna. (**c**) Flat AMC-backed antenna. (**d**) Bent AMC-backed antenna.

**Figure 15:**
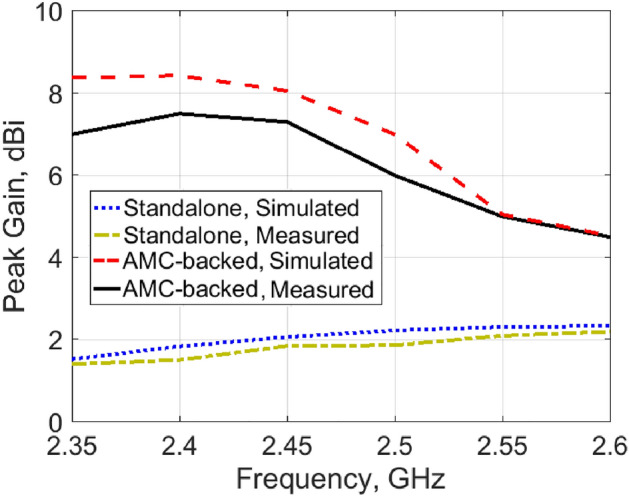
Simulated and measured peak gain of the proposed antenna in a flat state with and without AMC surface.

For free space measurements, the radiation characteristics of the fabricated antenna prototypes for the four different design cases aforementioned before were assessed in an anechoic chamber StarLab 18 as shown in Fig. 13. Comparison between simulated and measured radiation patterns in the *x*–*z* plane and *y*–*z* plane at the operating frequency of 2.45 GHz is shown in Fig. 14. It is clear that monopole-like radiation patterns are obtained for the antenna alone in both states. The AMC-backed antenna in both states has a directional pattern which is desirable for medical applications. Figure 15 shows the measured and simulated antenna gain of the flat antenna with and without the AMC surface. The measured data were determined by comparing them to that of a reference standard gain horn antenna. A measured peak gain of 7.2 dBi was achieved at 2.45 GHz for the antenna with the AMC surface compared to a peak gain of 1.9 dBi for the antenna alone, with a good agreement between the simulated and measured frequency responses.

**Figure 16:**
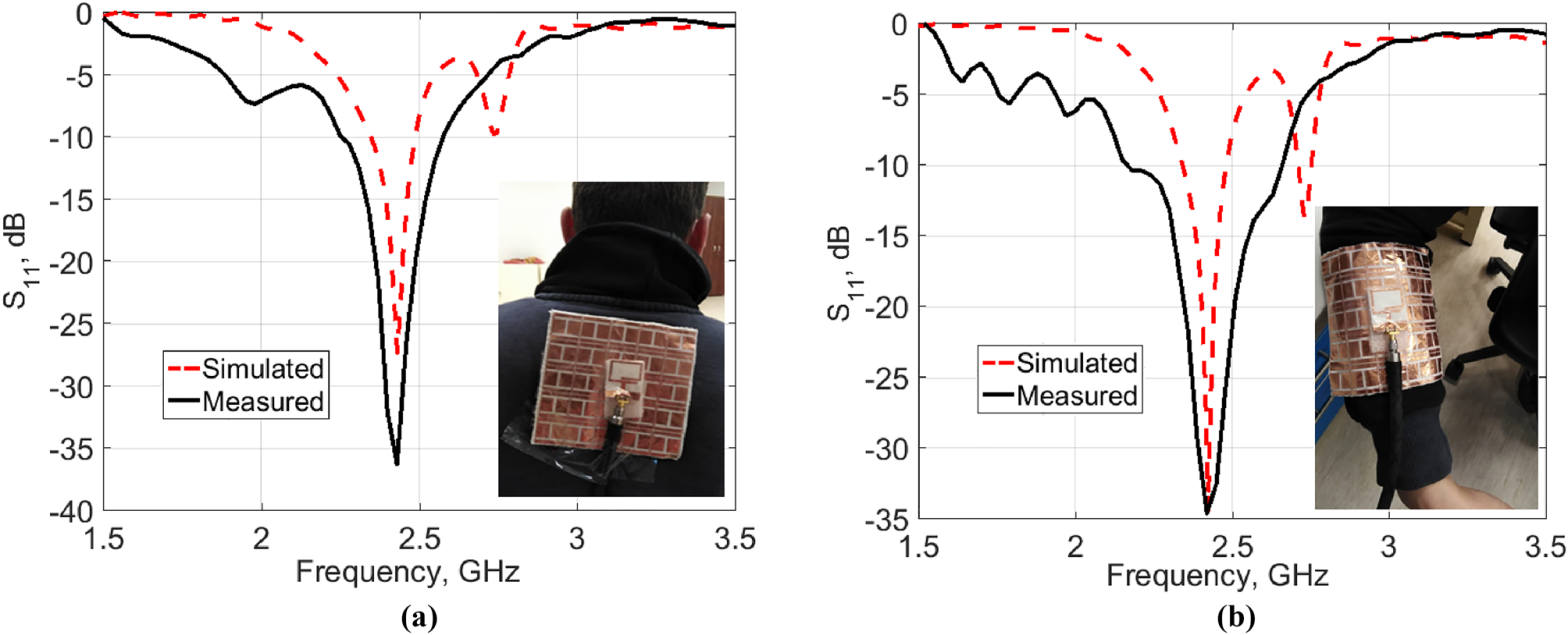
The |S_11_| responses of the fabricated AMC-backed antenna prototype placed close to an adult’s (**a**) back. (**b**) Arm of radius 50 mm.

For real human on-body measurements, a prototype of the antenna backed with the AMC surface was placed close to an adult’s back and arm. The measured |S_11_| responses along with the simulated ones are shown in Fig. 16a,b, respectively. Good performance is obtained for the antenna at two on-body placements, which validates the design strategy.

Table 1 compares the performance of the proposed single-band wearable antenna with state-of-the-art. Compared with the reported antennas of different substrates, the proposed integrated antenna features a good performance with all fabric-based substrates.

**Table 1:** Comparison with state-of-the-art.

Ref	Frequency (GHz)	Antenna size (mm^2^)	Antenna substrate/thickness (mm)	Reflector size (mm^2^)	Reflector-back antenna’s gain (dBi)	SAR (W/Kg)
^5^	4.8	27 × 34	Pellon/1.8	102 × 68	6.12	1.18,0.37
^22^	2.45	68 × 38	Rogers 5880 /1.57	68 × 38	6.88	0.244
^23^	2.4	30 × 20	denim material /0.7	46 × 46	7.8	0.013
^24^	1.8/2.45	124 × 90	Jean fabric /1	150 × 150	–	0.024/0.016
^25^	2.4	135 × 135	Polyester/0.1	135 × 135	8.5	0.07
^26^	2.45	46 × 46	Adopts denim/ 1	60 × 60	6.75	0.5
^27^	2.45/3.3	89 × 83	RO3003/1.52	89 × 83	6.2/3	0.29/0.29
^28^	2.4	50 × 50	Latex /1	50 × 50	0.12	0.714
^30^	2.4/5.8	–	Felt/2	85 × 85	–	–
^32^	2.45	–	Felt/3	50 × 50	2.22	0.0721
^33^	2.45	32 × 57	Pellon/3.6	124 × 124	4.6	0.166
^34^	2.45	30 × 45	Kapton polyimide/0.057	67.7 × 67.7	4.8	0.683
^35^	2.65	35.25 × 17.47	Adopt/1.5	39.4 × 33.4	2.99	1.25
^36^	2.45/1.57	85.5 × 85.5	Kevlar/5.62	85.5 × 85.5	1.94/1.98	0.78
^37^	7–28	60 × 50	Denim/0.7	–	–	0.171/0.52/0.69
This work	2.45	36 × 30	Cotton /0.9	122.5 × 122.5	7.2	0.18, 0.37

## Conclusion

In this work, a cotton-based wearable antenna convenient to be integrated with clothing was developed for 2.45 GHz wearable applications. The antenna was printed on a 0.9 mm pure cotton fabric (*ε*_*r*_ = 1.7) with a small overall size of 30 mm × 36 mm × 0.9 mm. To mitigate the body coupling effect, an AMC surface was integrated behind the antenna. The structural deformation of the integrated antenna was analyzed in free space as well as when it was placed in the vicinity of the human body, indicating its good suitability for operation when bent at both the *x*-axis and the *y*-axis. Further investigation, the SAR evaluation in the Voxel-based human body model considering flat and rounded body parts indicates that the integrated antenna provided average SAR values below the critical rate. The proposed design also features a directional pattern with respect to the on-body with high radiation characteristics, which makes it attractive for potential wearable applications.

## Data Availability

All data generated or analyzed during this study are included in this article (and there are no supplementary materials).
